# IRAK4 is essential for TLR9-induced suppression of Epstein-Barr virus *BZLF1* transcription in Akata Burkitt’s lymphoma cells

**DOI:** 10.1371/journal.pone.0186614

**Published:** 2017-10-31

**Authors:** Marc Jordi, Jeannine Marty, Vanessa Mordasini, Anna Lünemann, Scott McComb, Michele Bernasconi, David Nadal

**Affiliations:** 1 Laboratory for Experimental Infectious Diseases and Cancer Research of the Division of Infectious Diseases and Hospital Epidemiology and the Division of Oncology, University Children’s Hospital of Zurich, University of Zurich, Zurich, Switzerland; 2 Children’s Research Center, University Children’s Hospital of Zurich, University of Zurich, Zurich, Switzerland; University of North Carolina at Chapel Hill, UNITED STATES

## Abstract

Burkitt’s lymphoma (BL) is the most common childhood cancer in equatorial Africa, and is endemic to areas where people are chronically co-infected with Epstein-Barr virus (EBV) and the malaria pathogen *Plasmodium falciparum*. The contribution of these pathogens in the oncogenic process remains poorly understood. We showed earlier that the activation of Toll-like receptor (TLR) 9 by hemozoin, a disposal product formed from the digestion of blood by *P*. *falciparum*, suppresses the lytic reactivation of EBV in BL cells. EBV lytic reactivation is regulated by the expression of transcription factor Zta (ZEBRA), encoded by the EBV gene *BZLF1*. Here, we explore in the BL cell line Akata, the mechanism involved in repression by TLR9 of expression of *BZLF1*. We show that *BZLF1* repression is mediated upon TLR9 engagement by a mechanism that is largely independent of *de novo* protein synthesis. By CRISPR/Cas9-induced inactivation of TLR9, MyD88, IRAK4 and IRAK1 we confirm that *BZLF1* repression is dependent on functional TLR9 and MyD88 signaling, and identify IRAK4 as an essential element for TLR9-induced repression of *BZLF1* expression upon BCR cross-linking. Our results unprecedentedly show that TLR9-mediated inhibition of lytic EBV is largely independent of new protein synthesis and demonstrate the central roles of MyD88 and IRAK4 in this process contributing to EBV’s persistence in the host’s B-cell pool.

## Introduction

Primary infection with Epstein-Barr virus (EBV) is mostly asymptomatic, and more than 90% of the adult population worldwide carries the virus after it has established reversible latent infection [1,2]. This life-long, virtually harmless, host–virus coexistence must be regarded as the result of a long co-evolution based on modulation of EBV gene expression in different subsets of infected cells and the fine-tuned adaptation to the immune response of the human host [3]. Yet, EBV is associated with endemic Burkitt’s lymphoma (eBL), one of the most common childhood cancers in equatorial Africa, i.e., in areas where chronic co-infection with EBV and the malaria parasite *Plasmodium falciparum* prevails [4]. As a member of the gammaherpesvirus family, EBV establishes latency in B cells [5]. In eBL cells, EBV persists in a highly restricted form of latency [6], termed latency program I. In this program, EBV’s lytic and latent genes are repressed with exception of the EBV nuclear antigen (EBNA)1, which is essential for retention of EBV episomal genome in dividing cells. Thereby, the propagation of the virus to daughter cells is guaranteed, and the repression of EBV’s gene expression contributes to the evasion from the host’s immune system [7].

Latency of EBV is reversible, to ensure viral transmission to uninfected cells and to new hosts [2]. Thus, EBV periodically lytically reactivates, with the production of infectious viral particles and death of the infected B-cell. Lytic reactivation is set off by the expression of the immediate-early protein ZEBRA encoded by EBV’s master lytic gene *BZLF1*. ZEBRA is a transcription factor that induces a lytic cascade leading to early and late lytic EBV gene expression [8]. In BL cell lines latently infected with EBV (e.g. Akata cells), the lytic reactivation can be induced using diverse agents including phorbol esters (TPA), sodium butyrate (SB), transforming growth factor-β (TGF-β), and B-cell receptor (BCR) cross-linking anti-immunoglobulin G (anti-IgG) [9–13]. EBV particles and lytic proteins trigger a wide range of immune responses through innate immune mechanisms [14] and adaptive humoral [15] and T-cell responses [16]. Thus, the restriction to essential proteins required for the latent EBV persistence and replication is beneficial for both EBV and the host cell. To prevent aberrant spontaneous lytic reactivation, cell death and subsequent activation of the immune system, EBV latency is tightly controlled by histone modifications [17,18] and by DNA methylation [19]. In addition to these intrinsic regulatory factors, EBV can hijack the innate immune system, and, in particular, the signaling via Toll-like receptors (TLRs) to regulate the balance between latency and lytic reactivation [20].

Our group showed that activation of TLR9 signaling by hemozoin, a disposal product formed from the digestion of blood by *P*. *falciparum*, or by CpG ODNs suppresses the lytic reactivation of EBV in BL B-cells *in vitro* by affecting the histones state at the *BZLF1* promoter. We have shown that the TLR9-induced regulation of EBV lytic reactivation is not limited to the immediate early *BZLF1* mRNA expression but is also reflected on the Zta protein level, as well as on the immediate early *BRLF1*, early lytic *BXLF1* and late lytic *BXLF2* and *BCRF1* mRNA level. The activation of TLR9 significantly reduced EBV DNA copy numbers in the supernatant, indicating suppression of EBV release. Moreover, in this previous study we have shown that these mechanisms are not unique to the Akata Burkitt’s lymphoma cell line but also measurable in a Mutu I cell line derived from an African Burkitt’s lymphoma patient[21]. Nevertheless, the TLR9-induced mechanism involved in the lytic suppression remains largely unknown.

TLRs are essential elements of the innate immune system. They are transmembrane receptors involved in the recognition of pathogen associated molecular patterns (PAMPs) or danger associated molecular patterns (DAMPs), which initiate the inflammatory response by the production of cytokines [22,23]. Endosomal TLR9 is expressed in B cells and acts as a sensor for unmethylated CpG oligonucleotides (ODN) found on a large scale in bacterial DNA [24]. Upon stimulation, the TLR9 cytoplasmic Toll/interleukin-1 receptor (TIR) domain associates with the TIR domain-containing adaptor myeloid differentiation primary response gene 88 (MyD88). The latter recruits the interleukin-1 receptor-associated kinase (IRAK) 4 to TLR9 through interaction of the death domains of both molecules. IRAK-1 is activated by phosphorylation and associates with the TNF receptor associated factor (TRAF) 6, thereby activating the IκB kinase (IKK) complex, leading to activation of mitogen-activated protein (MAP) kinases (JNK, p38, MAPK) and of nuclear factor kappa B (NF-κB). NF-κB promotes the transcription of genes involved in cellular activation, proliferation and in the production of pro-inflammatory cytokines [25]. Recently, we showed that several elements of the TLR9 signaling pathway, including NF-κB, PI3K, ERK, JNK and p38, are not necessary for the inhibitory effect of TLR9 signaling on *BZLF1* mRNA expression [26]. Thus, additional investigation is required to precisely delineate how TLR9 signaling impacts on EBV lytic reactivation. Understanding the mechanisms favoring maintenance of lytic EBV infection could provide potential targets for treatments aiming at activating lytic EBV replication and inducing lysis of EBV-harboring cancer B cells.

Here, we aimed at advancing the detailed understanding of how TLR9 stimulation can suppress EBV lytic reactivation. First, by using protein synthesis inhibitors, we investigated whether inhibition of EBV lytic reactivation requires *de novo* protein expression, or if it acts through already existing elements. Next, we tested the importance of key components of the signaling pathway, which are directly downstream of TLR9 by generating Akata BL cells with either silenced or inactivated *TLR9*, *MyD88*, *IRAK4*, or *IRAK1* genes.

## Material and methods

### Cells and cell culture

The BL cell line Akata [12] was obtained from Dr. Andrew Bell (Birmingham, UK). Cells were grown in RPMI 1640 supplemented with 10% heat-inactivated fetal calf serum, streptomycin (100 mg/ml), penicillin (100 U/ml) and L-glutamine (2 mM). Akata cells transfected with the shLucGL3 (control) or shIRAK4 expressing plasmids were grown in complete medium supplemented with 200 μg/ml Zeocin (InvivoGen, Nunningen, Switzerland). Akata cells expressing a dominant-negative MyD88 (DN-MyD88 Akata) [26] were grown in the same medium supplemented with 0.4 mg/ml G418 (Promega, Dübendorf, Switzerland).

### Reagents and antibodies

ODN 2006 type B CpG oligonucleotide (# 11B15-MM) was bought from InvivoGen. The polyclonal rabbit α-human-IgG antibody (#A0423) was obtained from Dako (DakoCytomation, Zug, Switzerland). Cycloheximide (VWR international, Dietikon, Switzerland) was dissolved in H_2_O to a concentration of 10 mg/ml, whereas 4E1RCat (Sigma-Aldrich, Buchs, Switzerland) was adjusted to a concentration of 500 μg/ml in DMSO. Digitonin was dissolved in DMSO to a concentration of 20 mg/ml.

### EBV lytic reactivation and ODN CpG 2006 stimulation

Akata cells were resuspended at 1 x 10^6^ cells/ml in supplemented RPMI 1640 and stimulated with 0.5 mM end concentration ODN CpG 2006 (InvivoGen) 2 h prior to stimulation with 100 μg/ml anti-IgG (Dako). At 6 h after treatment with anti-IgG, cell pellets were harvested for RNA and protein extraction.

### Protein synthesis inhibition

For protein synthesis inhibition, Akata cells were treated with 4E1RCat (10 μM or 25 μM) or cycloheximide (33 μg/ml) for 30 min before ODN CpG 2006 treatment. Cells were then treated as described in the section above. The cells viability was assessed by Trypan Blue exclusion assay.

### hIL-10 ELISA

hIL-10 protein concentrations were determined in supernatants from stimulated cultures by standard capture ELISA (Ready- SET-Go, eBioscience, Vienna, Austria) according to the manufacturer’s instructions. Plates were read using a Synergy HT Multi-Detection Microplate Reader (BioTek, Luzern, Switzerland) at 450 nm and 570 nm. The values measured at 570 nm were subtracted from those of 450 nm and the cytokine concentration was determined by extrapolation from the standard curve.

### RNA preparation, reverse transcription and RT-qPCR (TaqMan)

RNA isolation, DNAse treatment, reverse transcription and quantitative PCR (RT-qPCR) was performed as described before [20,27]. *BZLF1*, *hIL-10*, *C-myc* (Hs00153408_m1; Life Technologies, Zug, Switzerland), *IRAK4* (Hs00211616_m1; Life Technologies) or *TLR9* (Hs00152973_m1; Life Technologies) mRNA expression was normalized to the mRNA of the housekeeping gene *HMBS* (same as above) resulting in Δ cycle threshold (ΔCT) values.

### Cell lysis and Western blot

Total protein lysates were obtained after lysing 10^6^ cells in RIPA complete buffer (50mM Tris-HCl pH 7.5, 150mM NaCl, 2mM EDTA, 1% NP40 complemented with 0.1% SDS, 1 x EDTA-free protease inhibitor cocktail (Roche, Rotkreuz, Switzerland)). Cell extracts were passed 10 times through a 25-G syringe. Protein content was determined using the Pierce BCA Protein Assay Kit (ThermoScientific, Zug, Switzerland), according to the manufacturer’s instructions. To analyze protein expression by western blot, protein (20 mg/well) was loaded into a NuPAGE 4–12% Bis-Tris Gel (Life Technologies), subjected to SDS-PAGE and transferred to a nitrocellulose membrane (GE Healthcare, Glattbrugg, Switzerland). The membrane was incubated with rabbit anti-IRAK1 (sc-7883, Santa Cruz Biotech, Santa Cruz, CA), rabbit anti-Akt (#9272), rabbit anti-pAkt (#4058), rabbit anti-Mek1/2 (#9122), rabbit anti-pMek1/2 (#2338), rabbit anti-Syk (#12358), rabbit anti-pSyk (#2710) or rabbit anti-β-actin (# 4967, all from Cell Signaling Technology, Allschwil, Switzerland) primary antibodies; subsequently with anti-rabbit (# 7074) or anti-mouse (# 7076) IgG HRP-linked secondary antibodies (Cell Signaling Technology). The signal was detected with the ECL Western Blotting Detection Reagents (GE Healthcare) and imaged using the LAS-3000 image reader (Fujifilm, Dielsdorf, Switzerland).

### EBV genome measurement

Genomic DNA was extracted from 2 x 10^6^ Akata cells using the QIAamp DNA Mini Kit (Qiagen, Hombrechtikon, Switzerland). Primers were diluted to a concentration of 300 nM with 150 ng DNA, 1x SYBR green MasterMix (#4309155, Life Technologies) and filled with water to a volume of 10 μl. Samples were measured in triplicates. Measurements were carried out on an ABI 7900HT Fast real-time PCR system (Applied Biosystems, Rotkreuz, Switzerland) and analyzed with SDS 2.2 software. SYBR green primers were as following:

*genomic BamH1 W*: forward: GCCAGACAGCAGCCAATTGT; reverse: GACTCCTGGCGCTCTGATG*; genomic HMBS*: forward: ACCAGCTCCCTGCGAAGAG; reverse: GAACTCCAGATGCGGGAACTT.

### CRISPR/Cas9 genome editing

**Fig 1:**
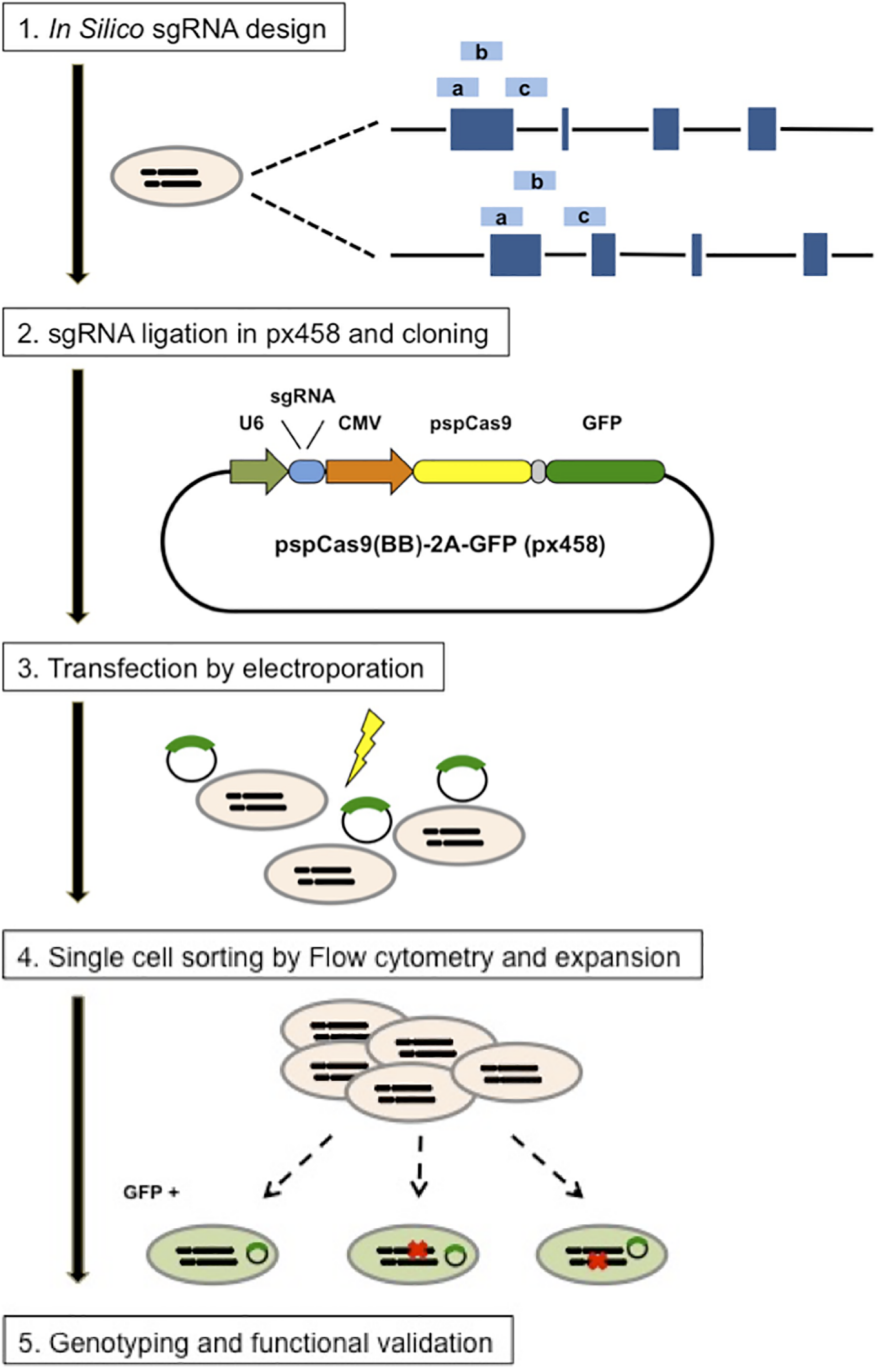
Experimental design for the establishment of CRISPR/Cas9 single cell clones. Steps for reagent design, construction, cell line expansion and characterization are depicted. Three different custom sgRNAs (light blue bars) were designed *in silico* via the CRISPR Design Tool (http://crispr.mit.edu/). sgRNA guide sequences were cloned into the expression plasmid pSpCas9(BB)-2A-GFP (PX458) bearing sgRNA scaffold backbone (BB), Cas9, and GFP. Cloned and sequence-verified pSpCas9(BB)-2A-GFP (PX458) plasmids were then electroporated into Akata cells, after 48h, GFP-positive cells were single cell sorted by FACS. Finally, the GFP positive single cell clones were expanded, genotyped and characterized. (Adapted from [28]).

Single guide RNA sequences targeting IRAK4, IRAK1, MyD88 and TLR9 were designed using the CRIPR Design webtool (http://crispr.mit.edu/). The three sequences having the lowest number of off-target sites were selected for each gene (Fig 1 and Table 1).

**Table 1:** CRISPR single guide RNA target sequences.

Target name	Genomic locus	Target Sequence (5’-3’)
IRAK4^-^ a	IRAK4 exon 2	AGGCAGCGCACATATGTTGATGG
IRAK4^-^ b	IRAK4 exon 2	TATGTGCGCTGCCTCAATGTTGG
IRAK4^-^ c	IRAK4 exon 2	GCCTCAATGTTGGACTAATTAGG
IRAK1^-^ a	IRAK1 exon 2	CGGTCTGGTCGCGCACGATCAGG
IRAK1^-^ b	IRAK1 exon 2	GATCAACCGCAACGCCCGTGTGG
IRAK1^-^ c	IRAK1 exon 2	GGTCTGGTCGCGCACGATCAGGG
MyD88^-^ a	MyD88 exon 1	GTTCTTGAACGTGCGGACACAGG
MyD88^-^ b	MyD88 exon 1	GCTCCAGCAGCACGTCGTCGCGG
MyD88^-^ c	MyD88 exon 3	ATGAAGGCATCGAAACGCTCAGG
TLR9^-^ a	TLR9 exon 2	CGCTGATGCGGTTGTCCGACAGG
TLR9^-^ b	TLR9 exon 2	ACTGGGTGTACAACGAGCTTCGG
TLR9^-^ c	TLR9 exon 2	GCTCACGGCTATTCGGCCGTGGG

Complementary oligonucleotides containing the guide RNA (without PAM sequence) and BpiI ligation adapter were synthesized by Microsynth (Balgach, Switzerland). The annealed oligos were ligated into BpiI digested pSpCas9(BB)-2A-GFP (px458) vector, a gift from Feng Zhang (Addgene plasmid # 48138). The sequence of the constructs was verified by DNA sequencing. CRISPR single cell clones were obtained by electroporation (Neon^®^ Transfection system, ThermoFisher Scientific, Zug, Switzerland) of Akata cells with the px458 plasmids. Transfected single cells, positive for green fluorescent protein (GFP), were sorted 48h after transfection using a FACS ARIA II cell sorter (BD Biosciences, Allschwil, Switzerland). Single cell clones were genotyped and characterized after expansion (Fig 1 and Table 2).

**Table 2:** Sequencing summary of CRISPR/Cas9 IRAK4^-^, TLR9^-^ and MyD88^-^ clones.

Clones	DNA sequence changes	Amino acid change
**IRAK4**^**-**^ **a3**	c.25inA	p.Thr9AspfsX19
**IRAK4**^**-**^ **a4**	c.24delA	p.Thr9HisX10
**TLR9**^**-**^ **b3**	c.2615_2690delCCTTCGTGGTCTTCGACAAAACGCAGAGCGCAGTGGCAGACTGGGTGTACAACGAGCTTCGGGGGCAGCTGGAGGA + c.2692T>C	p.Ala872GlyX73
**TLR9**^**-**^ **b5**	c.2672inC	p.Leu891ProfsX31
**TLR9**^**-**^ b6 1*	c.2672inC	p.Leu891ProfsX31
**TLR9**^**-**^ b6 2*	c.2447G>A + c.2655_2682delCTGGGTGTACAACGAGCTTCGGGGGCAG	p.Trp816X
**MyD88**^**-**^ **a3**	c.154_165delGTGCGGACACAG	p.53_56RTAVdel
**MyD88**^**-**^ **a5**	c.161delC	p.Thr54AspfsX46
**MyD88**^**-**^ **b1**	c.336delG	p.Asp112GlufsX12

DNA sequence changes nomenclature is based on the coding DNA sequence.

* TLR9^-^ b6 derives from two different clones whose sequence was determined by TA cloning and sequencing.

### DNA sequencing

Genomic DNA was extracted from 2 x 10^6^ CRISPR cell clones using the QIAamp DNA Mini Kit (Qiagen, Hombrechtikon, Switzerland). Each CRISPR target region on IRAK4, MyD88 and TLR9 were amplified by PCR using the following primers:

*IRAK4 Forward*: AGGAAGCAAACCCAGAGGAT;
*IRAK4 Reverse*: AACAGGGAACCACAGCAAAG
*MyD88 Forward*: GTCTCCTCCACATCCTCCCT
*MyD88 Reverse*: TTCCTTCCCATCTCCGCCTA
*TLR9 Forward*: TCAGCATCTTTGCACAGGAC
*TLR9 Reverse*: GCCCACAGGTTCTCAAAGAG

Primers were diluted to a final concentration of 300 nM with 500 ng DNA. PCR products were separated on an agarose gel for control. The remaining PCR products were purified using QiaQuick PCR purification kits (Qiagen, Hombrechtikon, Switzerland) and sent at Microsynth (Balgach, Switzerland) for sequencing.

Automated DNA Sequencers generate a four-color chromatogram showing the results of the sequencing run were then analysed with Geneious. When sequencing a PCR product derived from diploid genomic DNA, polymorphic positions will show both nucleotides simultaneously. A single peak position may have two peaks of different colors instead of just one indicating a SNP. Based on the chromatogram data analysis of the CRISPR/Cas9 knockout clones, SNP at the CRISPR/Cas9 mutation sites could be excluded.

### TA cloning

The sequencing chromatogram data of the TLR9^-^b6 clone showing some overlap indicating a polyclonal origin, the PCR products of were cloned, according to the manufacturer’s instructions, into a pCR2.1 backbone plasmid using the TA clone PCR Cloning Kit (Thermo Scientific). The transformation was performed using competent E. coli DH5 α cells. 20 bacterial ampicillin resistant colonies were selected. The plasmids that contained the PCR amplicon were extracted using a plasmid midi kit (Qiagen, Hombrechtikon, Switzerland). Plasmids were sent to Microsynth (Balgach, Switzerland) for sequencing.

### Statistics

Level of significance was evaluated by an unpaired Student’s *t* test or Tukey’s multiple comparison test (ANOVA) using Prism 6 (GraphPad Software, Inc.). P levels < 0.05 were regarded as statistically significant.

## Results

### TLR9-induced inhibition of *BZLF1* expression is largely protein synthesis independent

We have previously shown that TLR9-triggering by ODN CpG 2006 or Hemozoin inhibits EBV lytic reactivation, i.e. expression of *BZLF1*, induced by BCR cross-linking in BL cells *in vitro* [26]. Here, we aimed to elucidate the mechanism by which TLR9 inhibits *BZLF1* expression. In particular, we wanted to understand if *BZLF1* expression is inhibited directly, e.g. through post-translational modification of existing proteins induced by the TLR9 signaling, or indirectly, through *de novo* expression of proteins, e.g. of a transcription or repressor factor expressed upon TLR9 stimulation.

**Fig 2:**
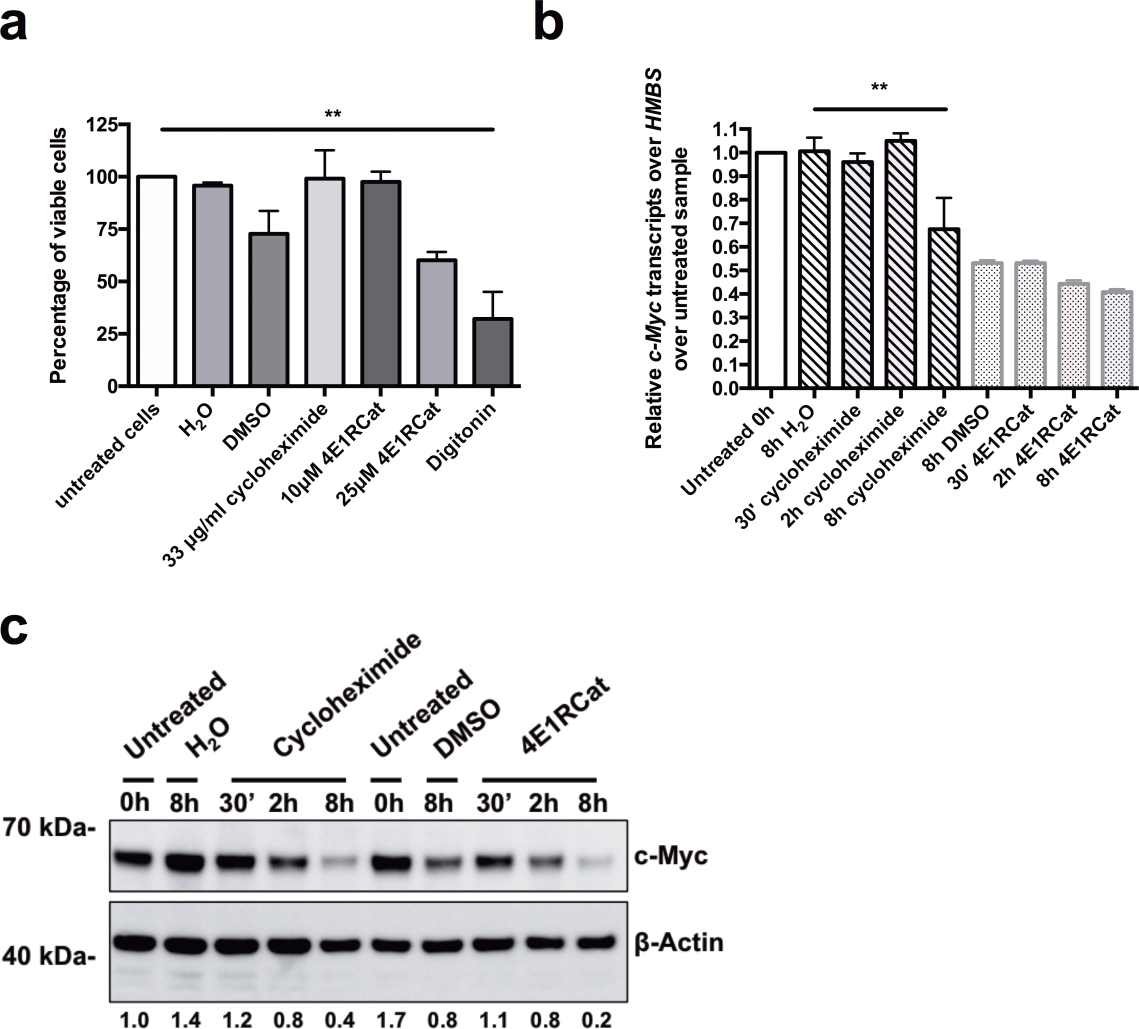
Treatment of BL cells with the mRNA translation inhibitors cycloheximide and 4E1RCat inhibits c-myc protein, but not *c-myc* mRNA expression. (**a**) Akata cells were treated with the protein synthesis inhibitors cycloheximide (33 μg/ml) or 4E1RCat (10 μM and 25 μM) for 8.5h. Cell viability was determined by Trypan blue exclusion assay. Untreated and vehicle-treated cells (H_2_O for cycloheximide and DMSO for 4E1RCat) were used as negative controls, and digitonin (30 μg/ml) treated cells as a positive control for cell toxicity. Results are shown as percentage of viable cells (n = 3). (**b**) and (**c**) Akata cells were treated for 8h with the protein synthesis inhibitors cycloheximide (33 μg/ml) or 4E1RCat (10 μM) for 30 min, 2h or 8h or with their vehicles, H_2_O or DMSO, respectively. (**b**) The *BZLF1* mRNA level relative to *HMBS* was measured by RT-qPCR and normalized to untreated samples. One representative experiment out of three is shown. (**c**) C-Myc protein expression level was measured by Western Blot. Densitometry of the c-myc protein expression was normalized over β-Actin expression and to untreated samples. Densitometry data of untreated cells was set to 1. Data are represented as mean ± SD. Statistics were calculated using the Tukey’s multiple comparison test (ANOVA). (*, *P*<0.05).

We treated Akata BL cells with a protein synthesis inhibitor, either cycloheximide or 4E1RCat. We chose these inhibitors because they act at the translational level and should not influence *BZLF1* mRNA levels. First, in order to determine the cytotoxicity of the protein synthesis inhibitors, we assessed the cell viability 8.5h after treatment of Akata BL cells with 33 μg/ml of cycloheximide, or 10 μM and 25 μM of 4E1RCat, respectively (Fig 2A). Treatment with 33 μg/ml cycloheximide, or 10 μM 4E1RCat did not affect cell viability, whereas 25 μM 4E1RCat or vehicle treatment, with the same volume DMSO as for 25 μM 4E1RCat, decreased cell viability compared to untreated cells to 60% (p = 0.554), and to 73% (p = 0.23), respectively. The treatment of the cells with digitonin, used as positive control for cell death, significantly reduced the cell viability to 30% (p = 0.0034) (Fig 2A).

To validate inhibition of protein expression, we measured expression of c-myc, which is highly expressed in Akata BL cells due to the chromosomal translocation t(8;14) [29]. Thus c-myc expression is a valid readout to confirm and validate mRNA translation inhibition in Akata cells. We incubated Akata cells with cycloheximide or 4E1Cat, and measured *c-myc* expression at the mRNA levels by RT-qPCR, and protein levels by Western Blot at various time points. The expression levels of *c-myc* mRNA were constant after 30’ and 2h treatment with cycloheximide, with relative *c-myc* transcripts of 0.96 (p = 0.9991) respectively 1.05 (p = 0.9994), whereas the level decreased to 0.68 (p = 0.0061) after 8h incubation with cycloheximide compared to control non-treated cells. The *c-myc* mRNA level remained stable after 30 min treatment with 10 μM 4E1RCat, compared to DMSO (p>0.9999). After 2h incubation, *c-myc* mRNA decreased by about 17% to 0.44 (p = 0.9458) and after 8h incubation by about 25% to 0.41 (p = 0.7510) relative *c-myc* transcripts compared to the *c-myc* mRNA level in DMSO-treated cells (Fig 2B). The *c-myc* protein levels clearly decreased in a time-dependent manner after 30’, 2h and 8h treatment with cycloheximide or 4E1RCat compared to the level measured in cells treated with their respective vehicle control (Fig 2C). Thus, despite a residual protein synthesis, we concluded that the protein inhibition was strong enough to allow us to investigate if TLR9-dependent inhibition of *BZLF1* expression acts directly through a post-translational mechanism or requires a *de novo* protein synthesis step.

**Fig 3:**
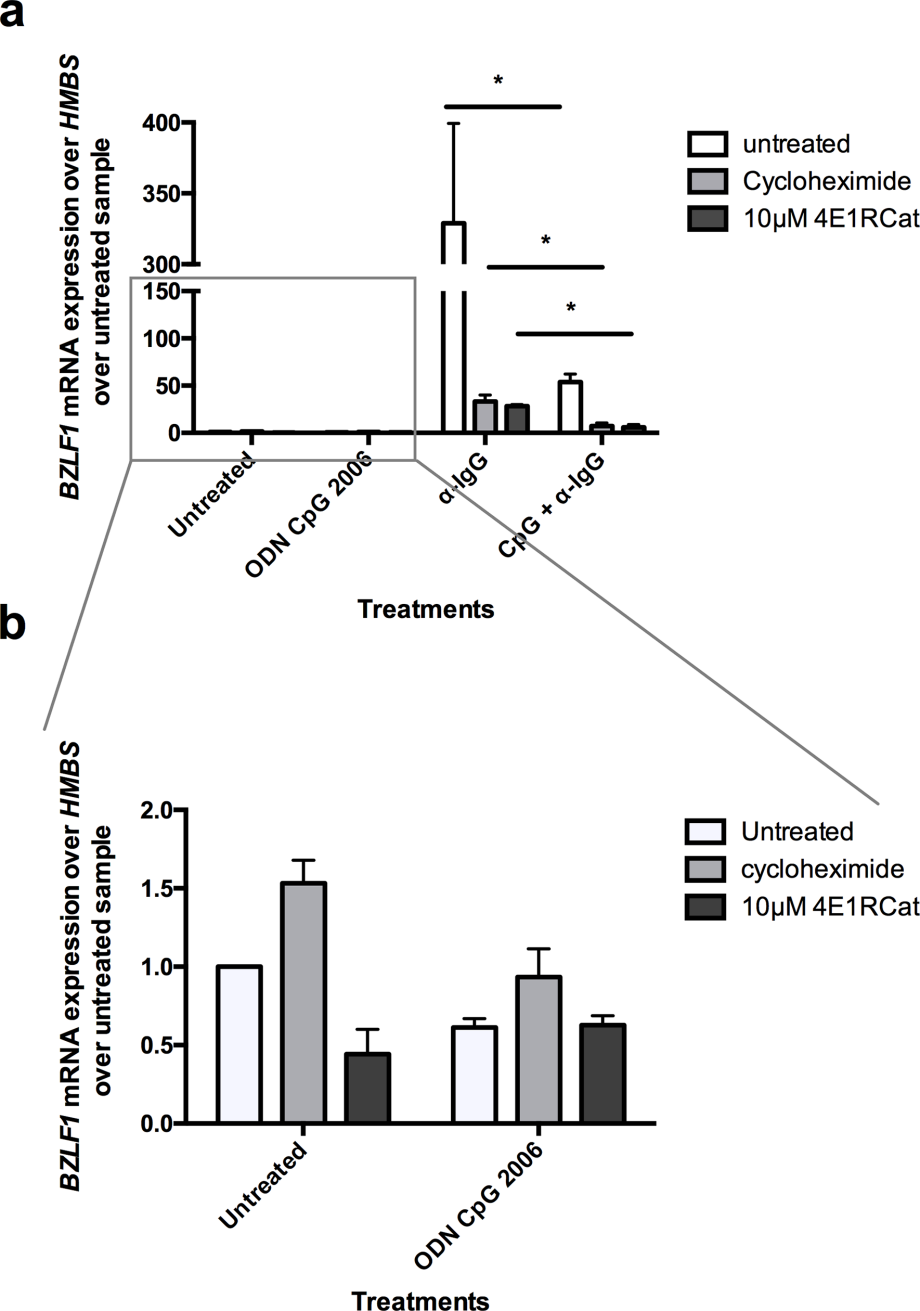
TLR9 triggering via ODN CpG 2006 inhibits EBV master lytic gene *BZLF1* mRNA expression despite partial protein synthesis inhibition. First, protein synthesis inhibitors cycloheximide (33 μg/ml) and 4E1RCat (10 μM) were added to Akata cells. After 30 min incubation, cells were treated for 2h with ODN CpG 2006, and finally for 6h with anti-IgG. After a total incubation time of 8.5h, cells were lysed and mRNA was extracted. The *BZLF1* mRNA levels relative to *HMBS* was measured by RT-qPCR and normalized to untreated samples (n = 3). (**b**) Enlargement of the *BZLF1* mRNA levels measured upon incubation with ODN CpG 2006, cycloheximide or 4E1RCat only presented in (**a**). Data are represented as mean ± SD. Statistics were calculated using the unpaired *t* test. (*, *P*<0.05).

Akata BL cells treated first for 30 min with protein synthesis inhibitors cycloheximide or 4E1RCat, after an additional treatment with ODN CpG 2006 or/and anti-IgG for 6h, EBV lytic reactivation was assessed by measuring the mRNA expression of the immediate early lytic transcription factor *BZLF1* (Fig 3A and 3B). Treatment with ODN CpG 2006, cycloheximide, or 4E1RCat alone had no effect on *BZLF1* mRNA expression levels (Fig 3B). In absence of protein synthesis inhibition, activation of TLR9 with ODN CpG 2006 inhibited the *BZLF1* mRNA increase after BCR cross-linking with anti-IgG by 83.7% (p = 0.0317) (Fig 3A). Treatment with cycloheximide or 4E1RCat led to a 14-times or 11-times lower *BZLF1* mRNA expression, respectively, after BCR triggering via anti-IgG compared to cells with no protein synthesis inhibition. This was expected, since expression of *BZLF1* upon BCR crosslinking is reinforced by a positive feedback loop exerted by ZEBRA, the protein expressed by the *BZLF1* gene, on its own promoter [30–32] (Fig 3A). Importantly, upon protein synthesis inhibition with cycloheximide or 10 μM 4E1RCat, TLR9 triggering via ODN CpG 2006 inhibited *BZLF1* mRNA increase by 77.7% (p = 0.0418) and by 78.7% (p = 0.011), respectively. In conclusion, taken into account all the technical limitations of these experiments, it seems that inhibition of *BZLF1* reactivation by TLR9 is largely mediated by a mechanism not requiring *de novo* protein synthesis.

### TLR9-induced inhibition of EBV lytic reactivation is MyD88, IRAK4 and IRAK1 dependent in Burkitt’s lymphoma cells

As shown above, inhibition of EBV lytic reactivation mediated by TLR9 activation is largely *de novo* protein synthesis independent. Previously, we have shown that the downstream TLR9 signaling elements NF-κB, phosphatidylinositol-3 kinase (PI3K), extra-cellular signal regulated kinase (ERK), *c-jun* N terminal kinase (JNK), and p38, are not fully responsible for TLR9-dependent *BZLF1* inhibition [26]. IRAK4 and IRAK1, which act immediately downstream of MyD88, were not tested. Thus, to know the importance of IRAK4 and IRAK1 proteins in TLR9-mediated inhibition of *BZLF1* expression, we used the CRISPR/Cas9 genome editing method [28] to inactivate IRAK4 and IRAK1, respectively. In addition, TLR9 and MyD88 were also inactivated to further confirm our previous results, and to use the TLR9 or MyD88 inactivated cells as positive controls.

**Fig 4:**
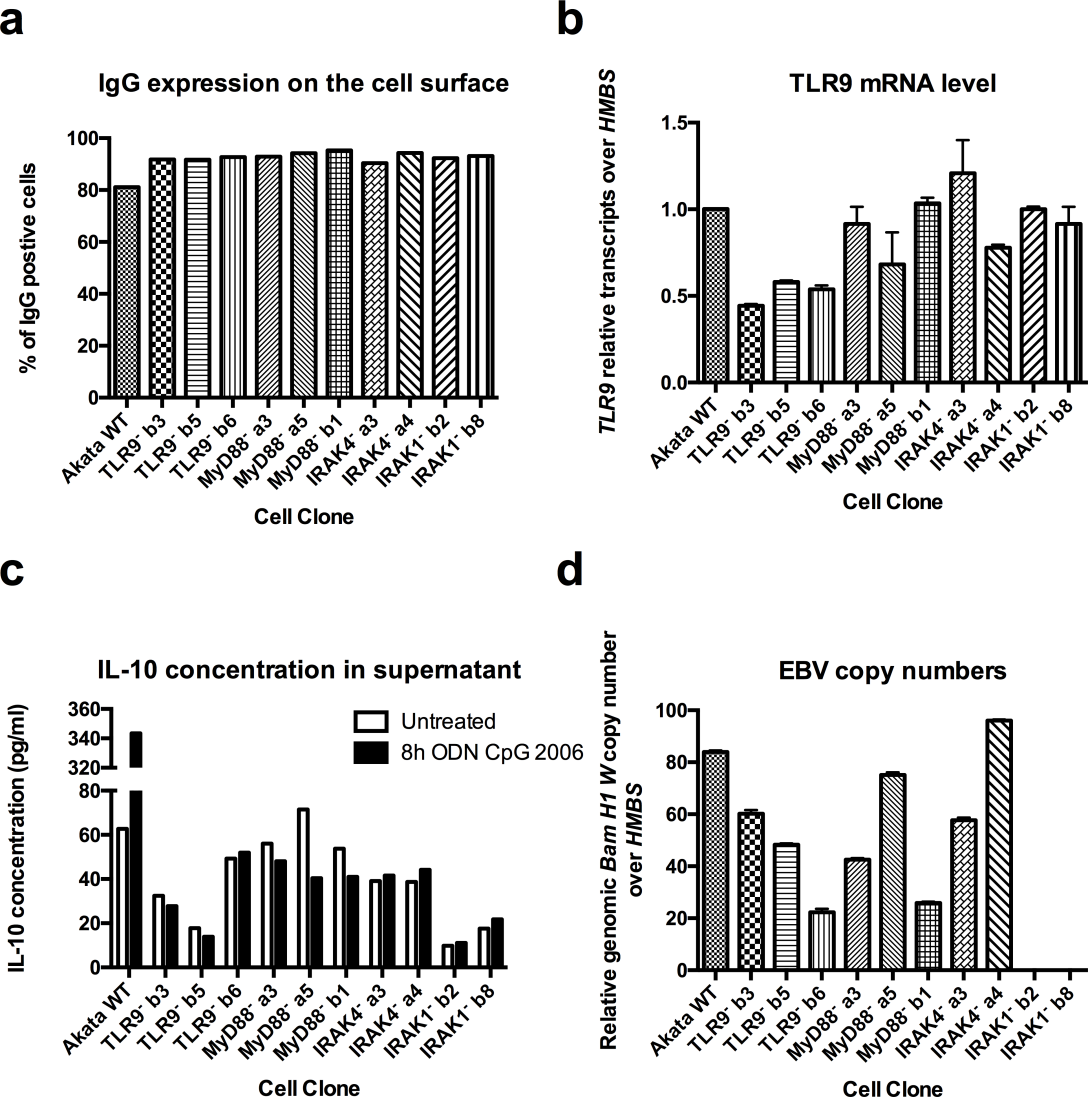
CRISPR/Cas9 inactivation of TLR9, MyD88, IRAK4 and IRAK1 results in a complete abrogation of TLR9 signaling. Akata Burkitt’s lymphoma cells transfected with px458 plasmids coding for Cas9 and for sgRNA targeting TLR9 (TLR9^-^b3, TLR9^-^b5 and TLR9^-^b6), MyD88 (MyD88^-^a3, MyD88^-^a5 and MyD88^-^b1), IRAK4 (IRAK4^-^a3 and IRAK4^-^a4) and IRAK1 (IRAK1^-^b2 and IRAK1^-^b8), respectively, were diluted for single cell cloning, sequenced and clones containing an early stop codon were selected for further characterization. (**a**) Percentage of IgG positive cells was measured by flow cytometry. (**b**) *TLR9* mRNA level was measured by RT-qPCR and normalized over *HMBS* and over WT Akata cells. WT Akata cells were set to 1. (**c**) IL-10 cytokine expression level measured by ELISA in the supernatant of untreated cells, and cells treated for 8h with ODN CpG 2006. (**d**) Viral *BamH1 W* copy numbers over cellular *HMBS* were determined by qPCR. (**a**) Has been performed once. (**b, c, d**) Shown is one representative experiment out of three. Data are represented as mean ± SD.

**Fig 5:**
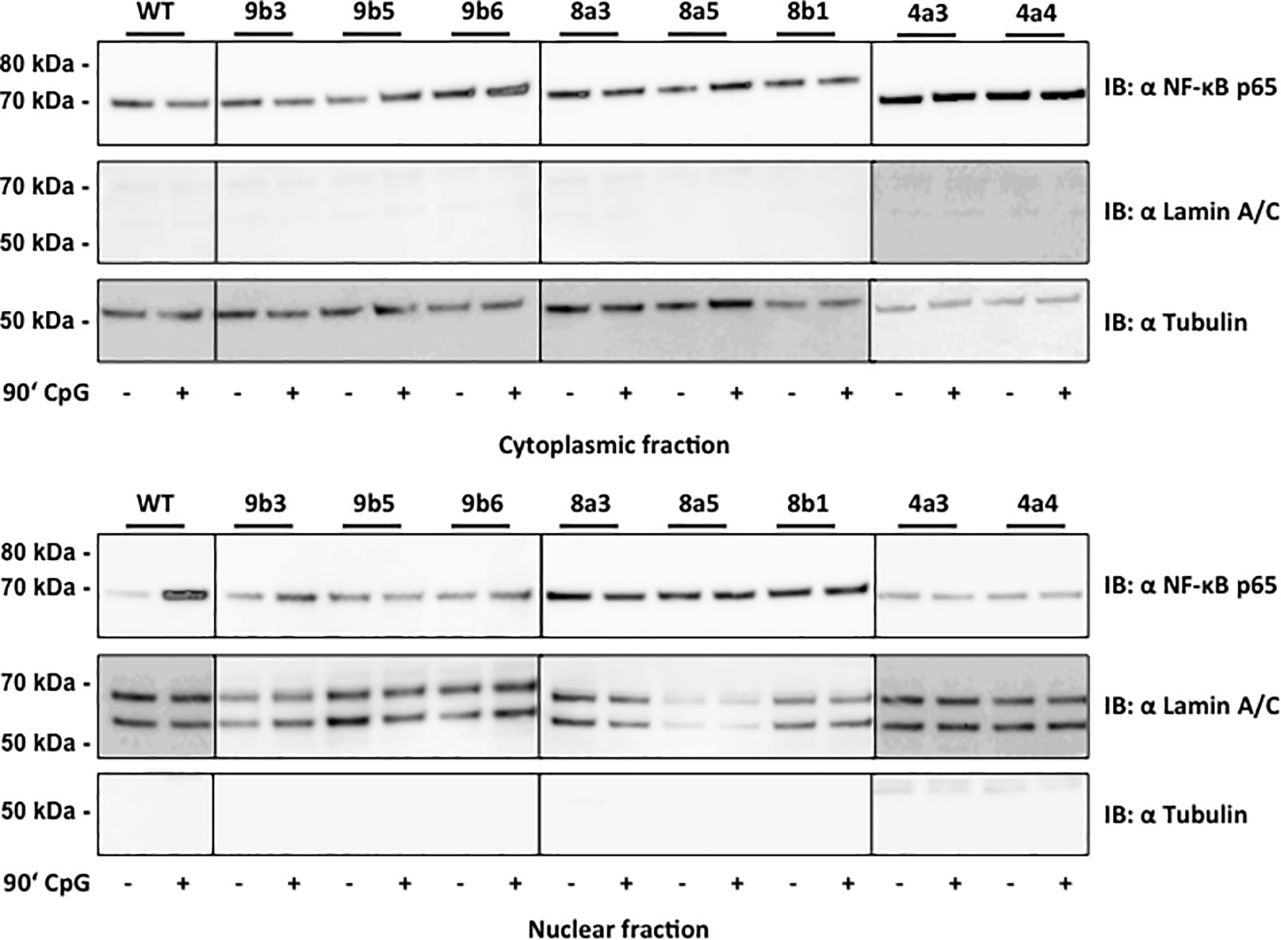
Inactivation of TLR9, MyD88 or IRAK4 inhibits NF-κB nuclear translocation upon treatment with ODN CpG 2006. Results show NF-κB p65 protein expression in the cytoplasmic and nuclear fraction from Akata WT cells and TLR9 (TLR9^-^b3, TLR9^-^b5 and TLR9^-^b6), MyD88 (MyD88^-^a3, MyD88^-^a5 and MyD88^-^b1) and IRAK4 (IRAK4^-^a3 and IRAK4^-^a4) CRISPR single cell clones. Cells were treated with ODN CpG 2006 for 1.5h. Lamin A/C and tubulin protein expression was measured as loading control for the cytoplasmic and nuclear fractions respectively. One representative experiment is shown out of three independent experiments.

After transfection of Akata cells with modified Cas9-gRNA-GFP (px458) expression plasmids (Fig 1), GFP-positive single cells were sorted and amplified. The clones were sequenced and 2–3 clones containing an early stop codon in the TLR9, MyD88, IRAK4, or IRAK1 coding sequences were selected for further characterization (Table 2). To verify that every cell clone had the same receptors expression level, IgG protein expression on the cells’ surface was characterized by flow cytometry (Fig 4A), and for TLR9 mRNA levels by were measured by RT-qPCR (Fig 4B). We then measured IL-10 protein levels in the supernatants (Fig 4C) and the NF-κB nuclear translocation (Fig 5), after 8h treatment with ODN CpG 2006, as readouts for the abrogation of the TLR9 pathway activity. Finally, we amplified the conserved EBV *BamHI W* sequence, present in multiple copies on the viral genome, by RT-qPCR to determine the EBV presence in the cell clones.

None of the inactivating mutations did affect the BCR surface expression levels of the cloned cells as determined by flow cytometry. 81.1% of the Akata WT cells expressed IgG on the surface, whereas 90.4% of the IRAK4^-^ a3, 94.3% of the IRAK4^-^ a4, 92.3% of the IRAK1^-^ b2, 93.1% of the IRAK1^-^ b8, 91.8% of the TLR9^-^ b3, 91.7% of the TLR9^-^ b5, 92.7% of the TLR9^-^ b6, 92.9% of the MyD88^-^ a3, 94.2% of the MyD88^-^ a5 and 95.3% of the MyD88^-^ b1 cell clones were IgG positive (Fig 4A). The *TLR9* mRNA levels in the clones ranged between 0.5-fold and 1.2-fold of the mRNA levels measured in the Akata WT cells (Fig 4B). On the other hand, the 5.5-fold IL-10 protein level increase measured after 8h ODN CpG 2006 treatment of WT Akata cells was not observed in the IRAK4^-^ a3, IRAK4^-^ a4, IRAK1^-^ b2, IRAK1^-^ b8, TLR9^-^ b3, TLR9^-^ b5, TLR9^-^ b6, MyD88^-^ a3, MyD88^-^ a5 and MyD88^-^ b1 cell clones, (Fig 4C) indicating that the inactivation of the TLR9 pathway was not a consequence of the variation in TLR9 mRNA levels. We confirmed the inactivation of the TLR9 pathway by measuring the NF-κB nuclear translocation by WB. After 90 min treatment with ODN CpG 2006, Akata WT cells showed a strong nuclear NF-κB protein levels increase compared to untreated cells, on the other hand in IRAK4^-^ a3, IRAK4^-^ a4, TLR9^-^ b3, TLR9^-^ b5, TLR9^-^ b6, MyD88^-^ a3, MyD88^-^ a5 and MyD88^-^ b1 cell clones the nuclear NF-κB was not increased by ODN CpG 2006 treatment (Fig 5). The selected cell clones IRAK4^-^a3, IRAK4^-^a4, TLR9^-^b3, TLR9^-^b5, TLR9^-^b6, MyD88^-^a3, MyD88^-^a5 and MyD88^-^b1 contained between 20 and 95 *BamHI W* copies per cell. For comparison, the Akata WT cells contained about 85 *BamHI W* copies (Fig 4D).

Despite variable EBV DNA copy numbers per cell, the detection of *BamHI W* copies allowed us to use the cell clones IRAK4^-^ a3, IRAK4^-^ a4, TLR9^-^ b3, TLR9^-^ b5, TLR9^-^ b6, MyD88^-^ a3, MyD88^-^ a5 and MyD88^-^ b1 for further *BZLF1* expression assays. By contrast and surprisingly, the *BamHI W* sequence was not detectable in IRAK1 cell clones IRAK1^-^ b2 and IRAK1^-^ b8, indicating that they had lost EBV genome. Since the cells were kept about 1 month in culture for the amplification step after single cell cloning. the transfection, selection and amplification steps were therefore repeated using IRAK1^-^b px458 plasmid to control that the loss of EBV copies in IRAK1 inactive clones was not due to the long culture time required for the amplification step, Akata cells transfected with a control px458 plasmid lacking a sgRNA target sequence, as well as untransfected Akata WT cells were isolated and amplified in parallel. The cells were directly tested for the TLR9 pathway activity by the measurement of IL-10 mRNA and proteins in the supernatant, as well as for the presence of EBV by the measurement of *BamHI W* copies (Table 3). Akata cells transfected with control px458 plasmid as well as untransfected Akata single cell clones all had an active TLR9 pathway and about 80% of the clones where EBV positive. This shows that the control px458 plasmid, the transfection, selection, and amplification steps necessary to obtain single cell clones had no influence on the TLR9 pathway activity and on the presence of EBV in the cells, which can be spontaneously lost. On the other hand, 55%, 40%, 50% and 60% of the IRAK1^-^, IRAK4^-^, TLR9^-^ and MyD88^-^ px458 transfected clones, respectively, had an inactive TLR9 pathway. Interestingly, of the clone with an inactive TLR9 pathway, 75%, 60% and 83% of the IRAK4^-^, TLR9^-^ and MyD88^-^ px458 transfected clones, respectively, were EBV positive (Table 3). In IRAK1^-^b px458 transfected cell clones, about 56% of the clones had an inactive TLR9 pathway, and 100% had lost EBV genome (Table 3). These results could suggest a role for IRAK1 in EBV viral persistence and transmission to dividing daughter cells.

**Table 3:** Summary of the CRISPR Cas9 transfection and characterization experiments.

	IRAK1^-^	IRAK4^-^	TLR9^-^	MyD88^-^	Akata px458-ctl
**Inactive TLR9 pathway (ko)**	13/23^a^	4/10^a^	5/10^a^	6/10^a^	0/12^a^
**EBV positive**	7/23^b^	6/10^b^	6/10^b^	9/10^b^	10/12^b^
**EBV positive / TLR9 ko**	0/13^c^	3/4^c^	3/5^c^	5/6^c^	0/0^c^

^a^ = number of single cell clones with an inactive TLR9 pathway over the total number of single cell clones analyzed

^b^ = number of EBV positive single cell clones over the total number of single cell clones analyzed

^c^ = number of EBV positive single cell clones over the number of single cell clones with an inactive TLR9 pathway.

Thus, as expected, after the sorting of a polyclonal cell population to single cell clones, differences in TLR9 and basal IL-10 mRNA expressions appear, as well as variation in the EBV DNA copy numbers between the single cell clones. Similar variability was also detected between single cell cloned cells transfected with a control CRIPSR/Cas9 plasmid, indicating that this was not unique to the knockout mutated cells. The reasons for this variability are hard to determine. However, determinant for this work is the complete abrogation of the TLR9 pathway activation, which was assessed by the IL-10 expression in the supernatant and the NF-κB nuclear translocation upon CpG treatment, and the ability of latent EBV to reactivate upon BCR cross-linking.

**Fig 6:**
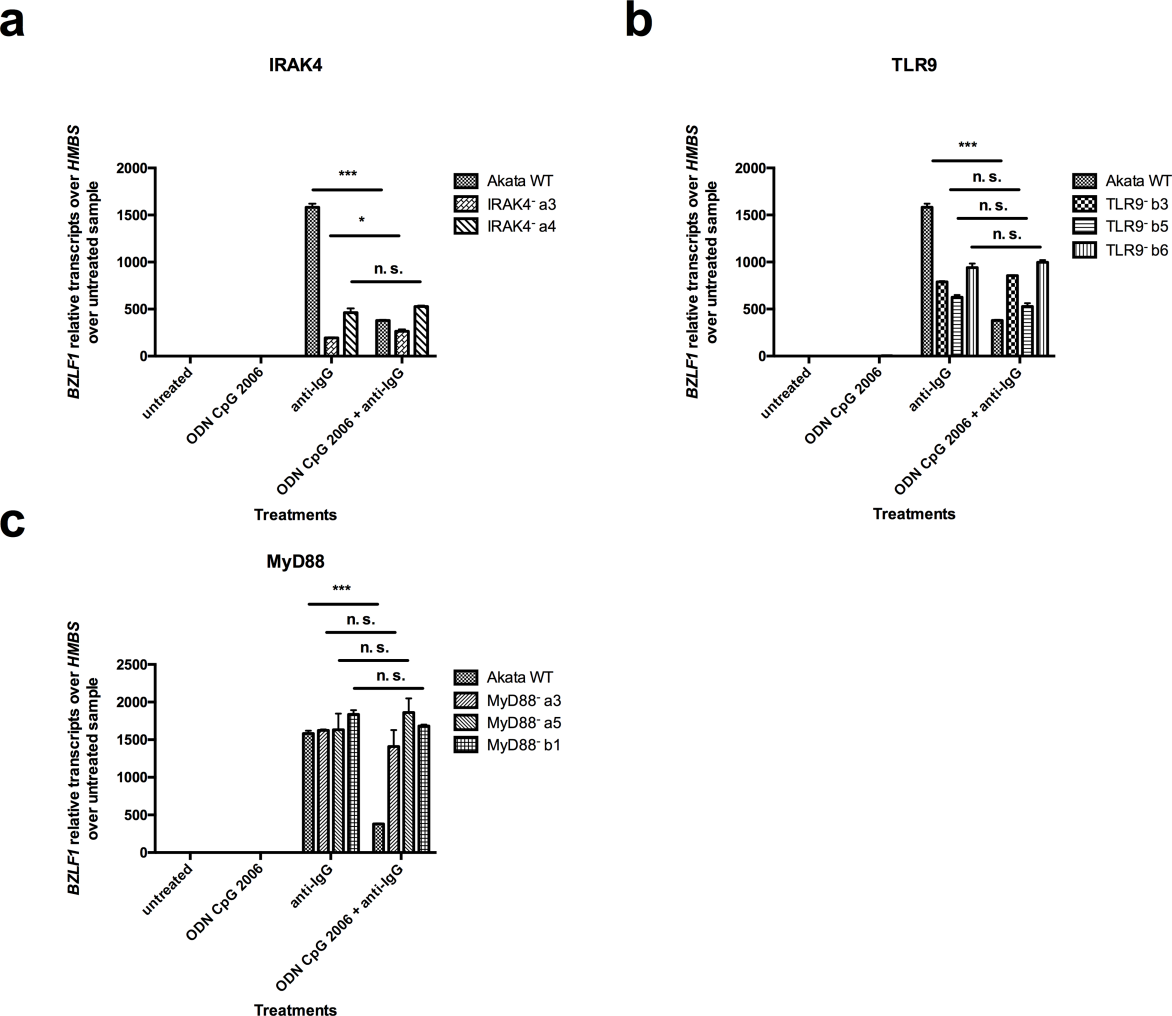
TLR9-induced inhibition of *BZLF1* expression is strictly IRAK4-, TLR9- and MyD88-dependent. CRISPR/Cas9 mutated IRAK4, TLR9 and MyD88 clones were treated for 2h with ODN CpG 2006 prior to lytic reactivation by Ig-crosslinking with anti-IgG for 6h. *BZLF1* mRNA expression level normalized to *HMBS* and relative to untreated cells was measured by RT-qPCR in IRAK4 clones, TLR9 clones and MyD88 clones. Shown is one representative experiment out of three. Data are represented as mean ± SD (n = 3). Statistics were calculated using the unpaired *t* test. (***, *P*<0.001; *, *P*<0.05; n.s., not significant).

Next, in order to determine the importance of IRAK4 in the TLR9-induced inhibition of *BZLF1* expression, the CRISPR/Cas9 mutated clones were treated with ODN CpG 2006 for 2h, followed by treatment with anti-IgG for 6h. TLR9 and MyD88 knockouts were used as controls. EBV lytic reactivation was determined by the measurement of *BZLF1* mRNA levels by RT-qPCR. For the IRAK4 inactive clones, IRAK4^-^ a3 and IRAK4^-^ a4, we measured 194 and 463 *BZLF1* relative transcripts over *HMBS* over untreated sample after BCR cross-linking, respectively (Fig 6A); and for the TLR9 inactive clones TLR9^-^ b3, TLR9^-^ b5 and TLR9^-^ b6, we measured 789, 627, and 940 *BZLF1* relative transcripts over *HMBS* over untreated sample after BCR cross-linking, respectively (Fig 6B). For comparison, for WT Akata cells we measured 1583 *BZLF1* relative transcripts over *HMBS* over untreated sample after BCR cross-linking. In the MyD88 inactive clones MyD88^-^ a3, MyD88^-^ a5 and MyD88^-^ b1 we measured 1624, 1632 and 1837 *BZLF1* relative transcripts over *HMBS* over untreated sample after BCR cross-linking, respectively (Fig 6C).

Thus, differences of lytic reactivation between the WT and the mutated knockout clones have been measured upon anti-IgG treatment. Those variations don’t correlate with EBV DNA copy numbers in the clones and raises the question whether the knockouts affect the BCR pathway and the lytic reactivation. However, the lytic reactivation that was measured upon BCR cross-linking was sufficient to asses that the TLR9-induced lytic inhibition was rescued by the TLR9, MyD88 and IRAK4 knockout mutations. Indeed, the mutated clones reached the same lytic reactivation level upon BCR cross-linking, whether TLR9 was activated or not. Although not surprising in the case of TLR9 mutation, this indicates the essential role of MyD88 and IRAK4 in the TLR9-induced lytic inhibition.

To explain the lytic reactivation differences we further assessed the spontaneous lytic reactivation and the BCR signaling pathway activity between the knockout clones.

The basal *BZLF1* mRNA levels measurements in untreated and ODN CpG 2006 treated cells show that the differences of spontaneous lytic reactivation between the TLR9-, MyD88- and IRAK4- CRISPR/Cas9 clones compared to WT Akata cells, are minor, although statistically significant for TLR9-b5, MyD88-a3 and MyD88-b1 clones (S1 Fig). Moreover, the differences in basal *BZLF1* mRNA levels do not correlate with the EBV DNA copies measured in each clones (Fig 4D) and neither with the EBV lytic reactivation upon BCR cross-linking (Fig 6). Spontaneous lytic reactivation is therefore, not the reason explaining neither the differences in EBV DNA loads nor the differences in *BZLF1* mRNA expression upon BCR cross-linking.

Next, in order to investigate if the lytic reactivation differences are due to an effect of the TLR9 and IRAK4 knockout mutations on the BCR signaling pathway, we stimulated the IRAK4^-^ a3, IRAK4^-^ a4, TLR9^-^ b3, TLR9^-^ b5 and TLR9^-^ b6 clones by BCR-crosslinking with anti-IgG for 15, 30, 60 and 120 minutes. The phosphorylation levels of Akt, Mek and Syk, three members of different branches of the BCR signaling pathway, were measured by western blot and compared with the phosphorylation levels in Akata WT cells. The activity of BCR signaling upon anti-IgG treatment was quantified by densitometric measurement of pAkt, pMek and pSyk levels normalized to total Akt, Mek and Syk protein levels, respectively. The results show only minor differences between the IRAK4^-^ and TLR9^-^ clones and the Akata WT cells in Akt, Mek or Syk phosphorylation levels upon treatment with anti-IgG (S2, S3 and S4 Figs). These results indicate that the differences in *BZLF1* mRNA expression upon lytic reactivation observed between Akata cells with inactivated IRAK4 or TLR9 and WT Akata are not due to a weakened BCR pathway activation.

The activation of TLR9 by ODN CpG 2006 in WT Akata cells reduced the expression of *BZLF1* mRNA by about 4.5-fold down to 380 relative transcripts (p = 0.0005) after BCR cross-linking. Interestingly, we showed that, despite differences in the *BZLF1* mRNA level after BCR cross-linking between the clones and WT Akata cells, the TLR9-induced reduction of *BZLF1* mRNA expression was completely rescued in the IRAK4^-^a3 and IRAK4^-^a4 clones with 264.6 (p = 0.0363) respectively 527.3 (p = 0.1855) *BZLF1* relative transcripts (Fig 6A); and in the TLR9^-^b3, TLR9^-^b5 and TLR9^-^b6 control clones, with respectively 855.5 (p = 0.0112), 527.2 (p = 0.078) and 998.1 (p = 0.2435) *BZLF1* relative transcripts (Fig 6B); and in the MyD88^-^ a3, MyD88^-^ a5 and MyD88^-^ b1 control clones with respectively 1408.6 (p = 0.2998), 1861.2 (p = 0.3735) and 1682.9 (p = 0.064) *BZLF1* relative transcripts (Fig 6C).

To sum up, by transfecting Akata cells with CRISPR/Cas9 plasmids containing sgRNA targeting IRAK4, TLR9 or MyD88 genes, insertions or deletions could be induced leading to an early stop codon in the target genes. In order to determine if the BCR and TLR9 crosslinking of the mutated single cell clones are comparable, the expression of these receptors has been measured. The characterization of the mutated clones showed that the surface IgG protein level remained stable and similar to the levels measured in WT Akata cells; that the TLR9 pathway activity was completely abrogated by the early stop codon in the target genes; and not as a consequence of the variation in TLR9 mRNA level; finally, despite variable EBV DNA copy numbers per cell, the IRAK4^-^ a3, IRAK4^-^ a4, TLR9^-^ b3, TLR9^-^ b5, TLR9^-^ b6, MyD88^-^ a3, MyD88^-^ a5 and MyD88^-^ b1 clones could be used for lytic reactivation assays.

In conclusion, these results show that in BL cells the TLR9-induced inhibition of lytic reactivation requires functional IRAK4, TLR9 and MyD88 proteins.

## Discussion

EBV lytic reactivation upon BCR cross-linking is inhibited by TLR9 stimulation with ODN CpG 2006 in B cells [26].

In the present work, for a matter of feasibility, we focused on the Akata cell line, which is a well-established model for EBV lytic reactivation. In our model we mimic the cross-linking of the BCR as it might occur in vivo, triggered by CIDR1α[33] or by an antigen, by treating the cells with anti-IgG. Moreover, by using ODN CpG 2006 we mimic the possible effects of hemozoin on TLR9.

First, we studied the importance of protein synthesis, and the involvement of the signaling proteins MyD88, IRAK4 and IRAK1, in this process. We found that (i) TLR9-mediated inhibition of EBV lytic reactivation in B-cells upon BCR cross-linking, is largely independent from *de novo* protein synthesis; (ii) the inactivation of either TLR9, MyD88, or IRAK4 completely abrogates the effect of ODN CpG 2006 on TLR9 signaling resulting in unhindered EBV lytic reactivation upon BCR cross-linking in the presence of ODN CpG 2006. Our results unprecedentedly show that TLR9 triggering activates a signaling pathway that does not only depend on protein synthesis to favor EBV latency in B cells upon stimulation of the BCR; and demonstrate the central roles of MyD88 and IRAK4 in this mechanism contributing to EBV’s persistence in the host’s B-cell pool.

Remarkably, activation of TLR9 by ODN CpG 2006 inhibited the transcription of EBV’s master lytic gene *BZLF1* in BL cells to a similar degree, even when mRNA translation was strongly inhibited. To direct our investigation, we tackled the question whether protein synthesis is decisive in EBV’s reactivation process upon challenges imposed to the host cells. In Akata BL cells the strong increase of ZEBRA expression upon BCR cross-linking, the protein coded by *BZLF1*, goes through a bi-phasic process that requires protein synthesis to be amplified [30]. Protein synthesis inhibitors are therefore expected to abrogate the positive feedback loop. In fact, upon BCR cross-linking, we observed lower *BZLF1* mRNA expression levels in cells treated with cycloheximide or 4E1RCat. Interestingly, TLR9 stimulation with ODN CpG 2006 inhibited *BZLF1* mRNA expression at the same ratio, whether protein synthesis was inhibited or not. This strongly suggests that the regulation of EBV’s lytic reactivation upon TLR9 stimulation with ODN CpG 2006 is, to a large extent, protein synthesis independent and suggests post-translational modifications to be responsible for EBV lytic gene repression. The question whether TLR9 activation also affects ZEBRA’s activity and the positive amplification ZEBRA exerts on its own promoter remains open. Our group revealed that TLR9 triggering affects histone modifications, which is, at least partly, responsible for EBV lytic gene repression [26]. In addition, several studies showed that epigenetic mechanisms including histone modifications or DNA methylation are responsible for the regulation of EBV lytic gene expression upon BCR cross-linking [34,35]. Consequently, even if we cannot completely exclude the existence of a newly synthesized unknown repressor factor(s), our results strongly indicate that EBV reinforces its latency by hijacking the TLR9-induced post-translational modifications to repress its lytic genes expression.

We unequivocally and unprecedentedly show that MyD88 and IRAK4 are essential for the TLR9-dependent inhibition of EBV lytic reactivation upon BCR cross-linking. This was possible after we inactivated *TLR9*, *MyD88*, or *IRAK4* genes in single cell clones by CRISPR/Cas9 methodology. Importantly, we proved that the canonical TLR9 signaling pathway was completely abrogated by the inactivating mutations as revealed by the lower IL-10 concentrations in the supernatant and the missing NF-κB nuclear translocation after stimulation with ODN CpG 2006. Of note, we excluded that *TLR9*, *MyD88*, or *IRAK4* inactivation affects the degree of BCR expression on the cell surface. Thus, the TLR9-induced abrogation of the effects subsequent to BCR-cross-linking is not due to changes in BCR expression degree.

Indeed, by inhibiting the lytic reactivation induced upon BCR cross-linking, TLR9 activation, via MyD88 and IRAK4, strengthens EBV’s latency.

Our findings contribute to an increased understanding of the mechanisms involved in the TLR9-induced inhibition of EBV lytic reactivation and confirm our previously reported observations [26]. Moreover, as reported earlier, TLR9-mediated suppression of EBV lytic gene expression is not restricted to latently infected BL cells but can also be observed *ex vivo* in primary cells upon EBV infection [20]. It seems likely that this feature is shared by other gammaherpesviruses, as we found that stimulation of TLR9, as well as of TLR7, similarly suppresses spontaneous and induced MHV-68 reactivation in mice [36]. In addition, NF-κB nuclear translocation was shown to suppress the lytic genes of the Kaposi's sarcoma-associated herpesvirus [37]. Thus, gammaherpesviruses seem to have evolved to exploit cellular signaling mechanisms in order to keep their latent state and avoid unnecessary aberrant lytic reactivation. The infectious viral particles produced upon EBV lytic reactivation are recognized by the host’s immune system and lead to an inflammatory response that could be harmful for both the virus and the host [38]. Our findings indicate that EBV developed a very strong and efficient strategy, by taking advantage of the host cells innate immune TLR9 signaling machinery through MyD88 and IRAK4, to ensure and maintain its reversible latency.

Most intriguingly, we failed to establish *IRAK1* mutated BL cell clones retaining EBV genomes. Since the 3 CRISPR plasmids we developed were efficient in mutating *IRAK1* gene in at least one single cell clone, this preliminary finding suggests that IRAK1 could play a central role in steering EBV persistence. EBV nuclear protein EBNA1 binds to the origin of viral replication and is essential to mediate replication and partitioning of its episome during the division of the host cell. EBNA1 function is regulated by the phosphorylation of ten specific sites [39]. The kinases responsible for EBNA1 phosphorylation have not been identified, but IRAK1 could be a potential candidate. Thus, one hypothesis for the loss of EBV in the *IRAK1*-mutated clones is that EBNA1’s function is altered by the inactivation of IRAK1, which affects the replication and partitioning of EBV’s genomic DNA in dividing cells leading to the dilution of the virus in the daughter cells and finally to its loss. However, it is important to consider that this result has only been observed in a limited number of clones, mostly transfected with the IRAK1^-^b CRISPR/Cas9 plasmid, the most efficient one, in three independent single cell cloning rounds. To assess the importance of IRAK1 activity for EBNA1 function it will be necessary to analyze more clones transfected with different IRAK1^-^ CRISPR/Cas 9 plasmids as well as further investigations.

In summary, our results demonstrate that IRAK4 is essential to inhibit EBV lytic reactivation upon TLR9-induction. Activation of the TLR9-MyD88-IRAK4 pathway activation favors EBV latency by post-translational modifications and is largely protein synthesis independent. Therefore, the TLR9-MyD88-IRAK4 pathway is a potential therapeutic target to support disruption of EBV latency in EBV-associated lymphoproliferative disorders.

## Supporting information

S1 Fig CRISPR/Cas9 mutated TLR9, MyD88 and IRAK4 clones were treated for 2h with ODN CpG 2006. *BZLF1* mRNA expression level normalized to *HMBS* was measured by RT-qPCR in TLR9^-^ clones, MyD88^-^ clones and IRAK4^-^ clones. Shown is one representative experiment out of three. Data are represented as mean ± SD (n = 3). Statistics were calculated using the unpaired *t* test. (***, *P*<0.001; *, *P*<0.05; n.s., not significant).
(TIFF)

S2 Fig Akata WT cells and TLR9 (TLR9^-^b3, TLR9^-^b5 and TLR9^-^b6) and IRAK4 (IRAK4^-^a3 and IRAK4^-^a4) CRISPR/Cas9 single cell clones were treated with ODN CpG 2006 for 15’, 30’, 60’ and 120’. (**a**) Results show pAkt and Akt protein expression. GAPDH or Tubulin protein expressions were measured as loading control. (**b**) Graphical representation of the densitometric measurement of (**a**). pAkt values were calculated relatively to the Akt level. One representative experiment is shown.
(ZIP)

S3 Fig Akata WT cells and TLR9 (TLR9^-^b3, TLR9^-^b5 and TLR9^-^b6) and IRAK4 (IRAK4^-^a3 and IRAK4^-^a4) CRISPR/Cas9 single cell clones were treated with ODN CpG 2006 for 15’, 30’, 60’ and 120’. (**a**) Results show pMek1/2 and Mek1/2 protein expression. GAPDH or Tubulin protein expressions were measured as loading control. (**b**) Graphical representation of the densitometric measurement of (**a**). pMek1/2 values were calculated relatively to the Mek1/2 level. One representative experiment is shown.
(ZIP)

S4 Fig Akata WT cells and TLR9 (TLR9^-^b3, TLR9^-^b5 and TLR9^-^b6) and IRAK4 (IRAK4^-^a3 and IRAK4^-^a4) CRISPR/Cas9 single cell clones were treated with ODN CpG 2006 for 15’, 30’, 60’ and 120’. (**a**) Results show pSyk and Syk protein expression. GAPDH or Tubulin protein expressions were measured as loading control. (**b**) Graphical representation of the densitometric measurement of (**a**). pSyk values were calculated relatively to the Syk level. One representative experiment is shown.
(ZIP)
