# Supplementary figures and images for: IRAK4 is essential for TLR9-induced suppression of Epstein-Barr virus BZLF1 transcription in Akata Burkitt’s lymphoma cells
Source: PLoS One. 2017 Oct 31;12(10):e0186614. doi: 10.1371/journal.pone.0186614 (PMC5663394; doi:10.1371/journal.pone.0186614)

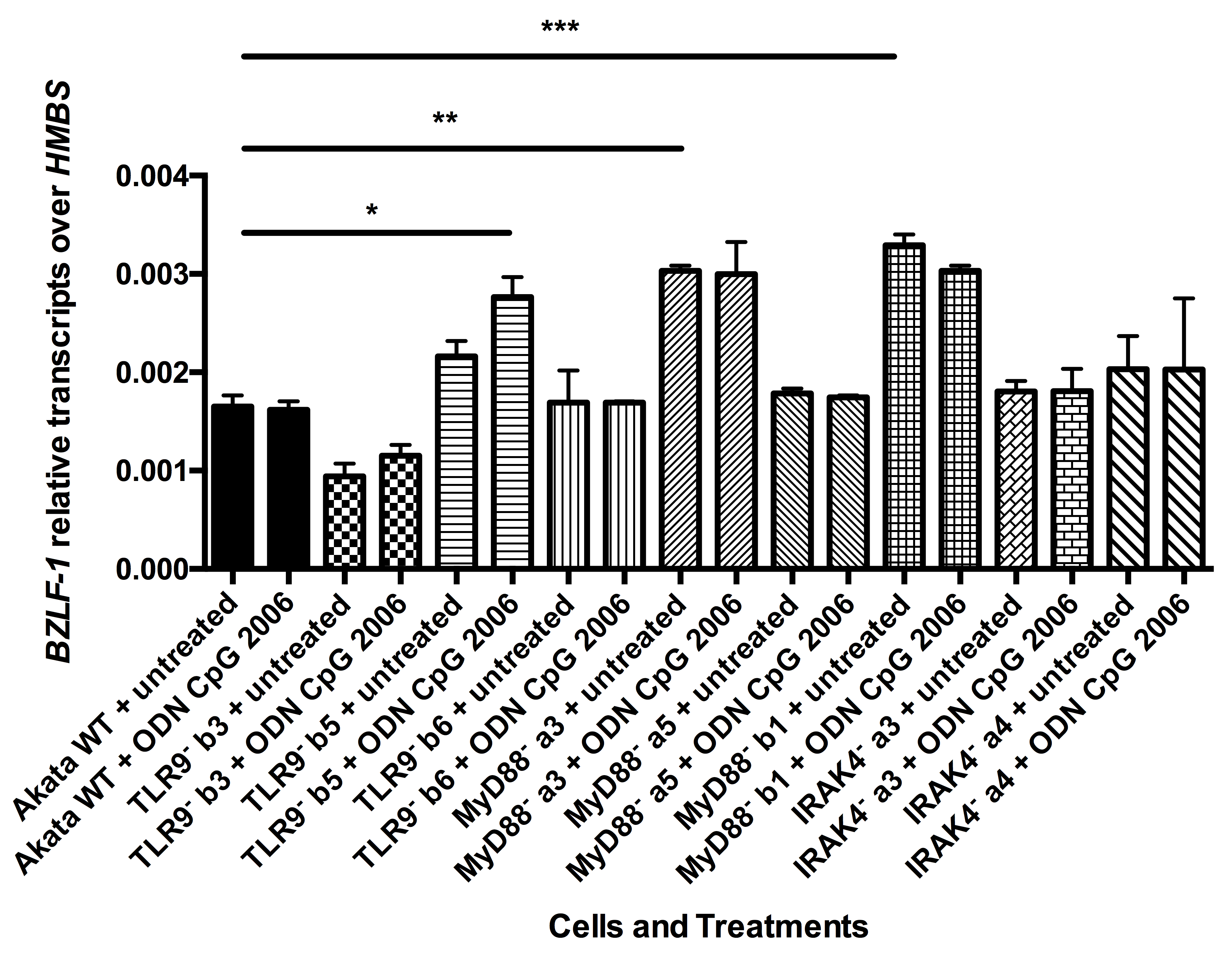

Supplement: S1 Fig — BZLF1 mRNA expression level normalized to HMBS was measured by RT-qPCR in TLR9- clones, MyD88- clones and IRAK4- clones. Shown is one representative experiment out of three. Data are represented as mean ± SD (n = 3). Statistics were calculated using the unpaired t test. (***, P<0.001; *, P<0.05; n.s., not significant). (TIFF) [file pone.0186614.s001.tiff]
